# Celiac Disease and Overweight in Children: An Update

**DOI:** 10.3390/nu6010207

**Published:** 2014-01-02

**Authors:** Antonella Diamanti, Teresa Capriati, Maria Sole Basso, Fabio Panetta, Vincenzo Maria Di Ciommo Laurora, Francesca Bellucci, Fernanda Cristofori, Ruggiero Francavilla

**Affiliations:** 1 Gastroenterology-Hepatology and Nutrition Unit, “Bambino Gesù” Children’s Hospital, Piazza Sant’Onofrio 4, Rome 00165, Italy; E-Mails: teresa.capriati@gmail.com (T.C.); msole.basso@opbg.net (M.S.B.); fabio.panetta@opbg.net (F.P.); francesca.bellucci@opbg.net (F.B.); 2 Gastroenterology Unit, Pediatric Clinic of University, Piazza Giulio Cesare 11, Bari 70124, Italy; E-Mails: fernandacristofori@gmail.com (F.C.); rfrancavilla@gmail.com (R.F.); 3 Epidemiology and Biostatistics Unit, “Bambino Gesù” Children’s Hospital, Piazza Sant’Onofrio 4, Rome 00165, Italy; E-Mail: vmaria.diciommo@opbg.net

**Keywords:** celiac disease, overweight, obesity, gluten free diet

## Abstract

The clinical presentation of celiac disease in children is very variable and differs with age. The prevalence of atypical presentations of celiac disease has increased over the past 2 decades. Several studies in adults and children with celiac disease indicate that obesity/overweight at disease onset is not unusual. In addition, there is a trend towards the development of overweight/obesity in celiac patients who strictly comply with a gluten-free diet. However, the pathogenesis and clinical implications of the coexistence of classic malabsorption (e.g., celiac disease) and overweight/obesity remain unclear. This review investigated the causes and main clinical factors associated with overweight/obesity at the diagnosis of celiac disease and clarified whether gluten withdrawal affects the current trends of the nutritional status of celiac disease patients.

## 1. Introduction

Celiac disease (CD) is a life-long condition that affects the small intestine in genetically susceptible individuals [1]. The global prevalence ranges from 1% to 2% [2,3]. In children, the symptoms upon CD presentation are highly variable and are influenced by age. Very young children often present with “classic” symptoms including diarrhea, abdominal distension, and growth retardation [4,5,6]. Diarrhea and malabsorption represent the typical presentation of CD in young children [7], while abdominal pain, vomiting, and constipation are atypical gastrointestinal symptoms more common in older children and teenagers. Furthermore, in children, CD can be diagnosed on the basis of the occurrence of extra-intestinal conditions such as arthritis, neurological diseases, and anemia [8,9] or on the basis of screening procedures in the absence of gastrointestinal symptoms (typical or atypical) and in child or adolescent with CD- associated conditions [10].

The presentation of CD has changed over time. In the last 2 decades, diarrhea and malabsorption have progressively decreased as the mode of CD onset among both adults and children, whereas atypical manifestations have increased. Interestingly, many reports indicate that CD can be associated with overweight or normal weight; hence, malnutrition is not always present at CD presentation [4,6,11,12]. Therefore, CD and obesity can coexist during both childhood and adolescence. After the first 2 cases reported by Semeraro [13] and Conti-Nibali [14] in 1986 and 1987, respectively, there have been several reports of the coexistence of CD and obesity/overweight in children and adolescents in the last 2 decades [15,16,17,18,19].

At present, in pediatric [20,21,22,23,24,25,26,27] and adult [28,29,30,31,32,33] case series of CD, the body mass index (BMI) at diagnosis is within the normal range in many patients. Nevertheless, the pathogenesis and clinical implications of the coexistence of CD and overweight/obesity remain unclear. The clinical relevance of this association is highlighted by the observation that CD patients with normal weight or overweight at diagnosis have a higher risk of developing obesity after starting a gluten-free diet (GFD), which definitely improves intestinal absorption in these patients. Moreover, the GFD regimen appears to be associated with high lipid and protein intake, particularly in adolescents [20,29].

The key studies concerning the pathogenesis and clinical evidence of the association between CD and overweight/obesity in subjects aged <18 years are discussed below. This review investigated the causes and main clinical factors associated with overweight/obesity at CD diagnosis. In addition, this review aims to clarify if gluten withdrawal affects the trend of the nutritional status of CD patients.

## 2. Clinical Evidence of CD and Overweight/Obesity

### 2.1. Summary of the Main Case Reports

The first pediatric case report [13] by Semeraro *et al*., in 1986 describes an obese 14-year-old girl who had been diagnosed with CD at the age of 1 year on the basis of a clinical condition characterized by malabsorption, diarrhea, and stunted growth (*i.e*., weight in the 7th percentile). The girl was started on a GFD, and had a normal weight at 2 years of age; however, she was overweight at 5 years of age and obese at 10. She had a negative family history for endocrine diseases and CD but a positive family history for obesity.

There are other reports of the development of obesity in children on a GFD who initially had malabsorption. For example Czaja-Bulsa *et al*. [15] describe the case of an 18-year-old boy with growth failure (*i.e*., <3rd percentile) and chronic diarrhea following gluten introduction and before CD diagnosis. However, after gluten withdrawal, his weight increased to the 97th percentile at 5 years of age despite persistent mucosal atrophy. More recently, Balamtekin *et al*. [19] reported a similar case of a 21-month-old child with the classic condition of malabsorption (*i.e*., chronic diarrhea, failure to thrive, and abdominal distension) at CD onset. After 11 years on a GFD, the child became obese (weight, >97th percentile).

Meanwhile, there are other reports of children with overweight/obesity at the time of CD diagnosis. The first published report describes a 5-year-old girl with obesity, short stature, and recurrent abdominal pain. The diagnosis of CD was suspected on the basis of family history, *i.e*., a sister with CD. A GFD attenuated the symptoms and improved height and weight growth [14]. Furthermore, in 2001, Franzese *et al*. [16] reported the case of a patient with steatohepatitis associated with obesity resistant to a low-calorie diet, in which CD was diagnosed on the basis of moderate persistent hypertransaminasemia. In 2006, Oso and Fraser [17] diagnosed CD in an obese teenager who had recurrent episodes of diarrhea, especially after eating spaghetti. At diagnosis, blood tests revealed low iron, GFD feeding normalized iron level, and the symptoms disappeared. However, the patient continued to gain weight (10 kg over 6 months) during follow-up. In 2009, Arslan *et al*. [18] reported the case of a 7-year-old obese patient with CD (weight, >95th percentile; weight/height ratio, 167%) suspected of having Hashimoto’s thyroiditis and affected by hypochromic anemia unresponsive to iron therapy. Moreover, Balamtekin *et al*. [19] describe the case of a 17-year-old obese girl with weight >97th percentile and a BMI of 32.9 with epigastric pain and vomiting. CD was diagnosed on the basis of the gastrointestinal symptoms, and the symptoms disappeared after a GFD was started. Nevertheless, her weight continued to increase.

### 2.2. Summary of Case Series

At present, few case series have been published on this topic. Valletta *et al*. [24] report the prevalence of overweight (BMI *z*-score > +1) and obesity (BMI *z*-score > +2) to be 11% and 3%, respectively, in 149 children newly diagnosed with CD between 1991 and 2007. The authors found that after initiating a GFD, the BMI *z*-score increased significantly and the percentage of overweight subjects almost doubled. In a retrospective study, Venkatasubramani *et al*. [22] report 5% of patients had a BMI > 95th percentile among 143 patients with CD diagnosed between 1986 and 2003. Among the obese patients, the most common symptoms at onset were abdominal pain, diabetes, and diarrhea.

Brambilla *et al*. [25] compared 150 children with CD on a GFD with 288 healthy sex- and age-matched children. They also retrospectively evaluated changes in BMI from CD diagnosis to the last clinical evaluation. The median BMI of CD patients was significantly lower than that of the healthy controls. In particular, children with CD were less frequently overweight or obese (12% *vs*. 23.3%) and more frequently underweight (16% *vs*. 4.5%) than the controls. However, after GFD feeding, the number of underweight subjects decreased significantly, while the number of overweight subjects increased slightly.

Reilly *et al*. [26] studied 142 children with newly diagnosed CD from 2000 to 2008. Nearly 19% of patients had a high BMI at diagnosis (12.6% overweight and 6% obese), while 74.5% had a normal BMI. Meanwhile, the BMI of 75% of the patients with high BMI at diagnosis decreased on a GFD. Among patients with a normal BMI at diagnosis, weight *z*-scores increased significantly after diet treatment and 13% became overweight. Interestingly, in that survey, the initial symptom in 28% of overweight CD patients was abdominal pain and the diagnosis was made on the basis of the screening test in a asymptomatic portion of the population by 28%. Venkatasubramani *et al*. [22] also found abdominal pain is one of the most common features of CD presentation in overweight patients. Another important aspect of their survey results is that the CD diagnosis was made on the basis of the screening test in at least 25% of overweight patients. Brambilla *et al*. [25] suggest that identifying CD patients on the basis of screening tests, and not symptoms, may increase the probability of finding overweight or obese subjects at CD diagnosis.

In a cross-sectional multicenter study, Norsa *et al*. [27] enrolled 114 children with CD in serologic remission, who were on a GFD for at least 1 year. The anthropometric measurements at diagnosis revealed that 9.6%, 76.3%, 8.8%, and 5.3% were underweight (BMI < 5th percentile), had normal weight (BMI = 5–85th percentile), were overweight (BMI = 85–95th percentile), and were obese (BMI > 95th percentile), respectively. After gluten withdrawal, the prevalence of overweight and obesity increased to 11.4% and 8%, respectively.

In a prospective case–control study, Barera *et al*. [34] found reduced fat mass, decreased bone mineral content, and lower lean body mass in the limbs of 29 children newly diagnosed with CD compared to healthy controls; all patients were normalized (*i.e*., approaching corresponding parameters in the control population) on a GFD. Table 1 summarizes the main results of the abovementioned reports.

**Table 1:** Prevalence of overweight/obesity in CD.

Author (Year)	Country (Sample Size)	Overweight/Obesity at Presentation (%)	Overweight/Obesity after Initiating a GFD (%)	Reference
Aurangzeb (2010)	Australia & New Zealand (*n* = 25)	20.8/0	ND/ND	[21]
Venkatasubramani (2010)	Milwaukee, WI, USA (*n* = 143)	ND/5	ND/3	[22]
Balamtekin (2010)	Ankara, Turkey (*n* = 220)	ND/0.5	ND/ND	[23]
Valletta *et al*. (2010)	Italy (*n* = 149)	11/3	21/4	[24]
Reilly *et al*. (2010)	NY, USA (*n* = 142)	12.6/6	20/4	[26]
Norsa *et al*. (2011)	Italy & Israel (*n* = 114)	8.8/5.3	11.5/8.8	[27]
Brambilla *et al*. (2011)	Italy (*n* = 150)	11.3/0.7	9.4/0	[25]

GFD: gluten-free diet; ND: not done.

A clarification regarding the methodology of these studies should be made: in adults and children, the main criterion for defining overweight/obesity is BMI (or Quetelet index), which is calculated by dividing weight (in kg) by height (in m) squared. BMI is an expression of the weight “adjusted” to stature and is an index of adiposity; it is most strongly correlated with body fat and less correlated with stature. Despite its limitations, BMI is easy to calculate and widely used, especially in large-scale studies, to assess the risks of diseases. The internationally accepted age- and sex-standardized threshold values of BMI for nutritional status in adults are those proposed by the World Health Organization [35]. However, the curves of children’s weight and height vary with growth, following development during puberty (with its consequences on body composition), and sex. Therefore, references for different age groups (*i.e*., the distribution of percentiles with cut-off points) are necessary. There are many different percentile tables based on data from reference populations that also have very different anthropometric characteristics. Ideally, the study population should be compared with tables based on national curves. Alternatively, the International Obesity Task Force (IOTF), which is the main organization of childhood obesity scholars, have validated tables with mean percentiles derived from cross-sectional studies of different populations (e.g., the USA, Brazil, Hong Kong, Singapore, Holland, and Great Britain) to enable international comparisons [36].

In this regard, the abovementioned studies have a discrete methodological heterogeneity. Although all are based on the calculation of BMI, they used different categorizations in various case series, such as the BMI percentile, BMI *z*-score, and IOTF cut-off point. In addition, different studies were conducted on geographically diverse populations, and only a few studies compared the case population with a control population [21,25].

## 3. Pathogenetic Link between CD and Overweight/Obesity

### 3.1. Overweight and Obesity in Newly Diagnosed CD Patients: The “Compensatory” Hypothesis

Semeraro first hypothesized that the atrophy of the duodenum—jejunum in CD patients could be compensated by enhanced absorption in the distal intestinal segments [13]. The fat absorption coefficient could in fact be preserved in a patient with a partially atrophic bowel [13]. This process could be similar to that occurring in the residual bowel after surgical resection, which involves structural changes that lead to an increased absorptive attitude of the intestine. The intestinal adaptation consists of morphological changes of the mucosa, including increased villus height, crypt depth, and epithelial cell number. In CD patients, atrophy determines the loss of normal intestinal function. This can hypothetically induce increased absorption of the functionally preserved intestinal tract. If this process overcompensates, it could lead to the extraction of energy exceeding the child’s needs, thus increasing the risk of overweight/obesity [13].

This compensatory hypothesis appears to be supported by some of the first published cases of adolescents affected by CD who continued to present with overweight or obesity despite persistent villous atrophy on jejunal biopsies [14,15]. The compensatory surface area of the small intestine appears to increase with patient age. Therefore, the intestine may develop the ability to absorb an adequate amount of compensatory energy [13]. This notion is corroborated by the particular distribution of symptoms upon CD diagnosis, which appears to be related to age [5,6,7]. Children aged less than 2 years often exhibit the classic CD presentation, which includes malabsorption. In contrast, older children, adolescents, and adults often present with atypical symptoms. This appears to be concordant with the compensatory hypothesis. In fact, the classic symptoms may be due to a lack of intestinal adaptation, which is less developed in young children as mentioned above. The absence of intestinal adaptation induces the occurrence of severe and classic symptoms including malabsorption and celiac crisis, which can be found in very young children newly diagnosed with CD. As intestinal adaptation is a time-dependent phenomenon, the probability that an individual’s mucosa is modified increases with age. Therefore, CD symptoms could be attenuated in older children and adolescents.

Concordant with this hypothesis, there is no correlation between the presentation of CD and the degree of villous atrophy [37] or the extent of the intestine involved as visualized through video-capsule endoscopic procedures [38]. The morphological appearance of the mucosa may be unrelated to its functional expression responsible for the severity of the presenting symptoms.

In addition, the nutritional status of the underlying population is clearly very important for the correct interpretation of BMI in children with CD at diagnosis. CD may indeed develop in patients with overweight/obesity, reflecting an individual’s predisposition (*i.e*., genetic, nutritional, and environmental factors). The worldwide prevalence of overweight/obesity in children has increased over the last 2 decades; an estimated 60 million children will be overweight or obese by 2020 [39]. In this scenario, the symptoms of malabsorption that could manifest in overweight patients at CD onset may reduce the prevalence of overweight/obesity in CD patients compared to the reference population but increase it in comparison to what is usually expected in CD patients.

### 3.2. The Effect of Gluten Withdrawal on Overweight/Obese CD Patients

Overweight or obesity may develop in CD patients after gluten withdrawal. The main surveys on children discussed above report the normalization of BMI in underweight and overweight patients on a GFD, although they also report the development of overweight and obesity independent of baseline nutritional status [21,22,23,24,25,26,27,34]. In consideration of the abovementioned “compensatory” hypothesis, it can be supposed the mucosal healing following gluten withdrawal is responsible for the normalization of BMI in both underweight and overweight patients as a result of the recovery of energy balance. Therefore, the restoration of the absorptive functions of the whole bowel could constitute a physiological redistribution of the absorptive attitude in whole bowel mucosa. This could result in an increased energetic yield in patients with symptoms of malabsorption. However, in patients with a mucosa adapted to supply a higher energetic yield, the improved absorptive function of the whole bowel could induce the normalization of caloric balance. Nevertheless, it remains to be determined if a GFD itself is a cause of the development of overweight/obesity in CD patients.

The unpalatability of some gluten-free foods may induce a preference toward hyperproteic and hyperlipidemic foods [20,29,40]. This may consequently lead to increased energy intake followed by excessive weight gain [41]. Mariani *et al*. [20] examined the eating habits and diet composition of 47 adolescents with CD and compared them to those of 47 healthy age-matched control subjects. They divided the CD patients into 2 subgroups according to compliance with a GFD: group 1A patients rigorously adhered to a GFD, while group 1B patients did not comply with a GFD. Compared to Recommended Dietary Allowances, total energy, lipid, and protein intake were higher and carbohydrate intake was lower in CD patients and controls. Total caloric intake and lipid and protein consumption were higher in group 1A than in group 1B. As a consequence, overweight/obesity was more frequent in group 1A (72%) than in group 1B (51%) and the controls (47%).

Several studies confirm long-term GFDs may not be nutritionally balanced. Indeed, there is clinical evidence indicating high simple sugar, protein, and saturated fat intake as well as low complex carbohydrate and fiber intake in such diets [20,42,43]. Concordant with this pediatric evidence, higher total caloric [44], carbohydrate, and fat [45] intake is reported among adults with CD than among healthy control subjects. In contrast, a few studies in adults [46,47] and children [48] report reduced caloric intake in CD patients on a GFD.

Besides increased total caloric intake, the macronutrient composition of the diet may be involved in the pathogenesis of overweight and obesity in patients with CD. Carbohydrates are the major energy source in the diet of children in developed countries and are the dietary components that most strongly affect blood glycemia. Both the quantity and type of carbohydrates are the determinants of postprandial glycemia [49]. The glycemic index (GI) is a parameter used to classify foods according to their postprandial glycemic response [50].

Many gluten-free foods are characterized by a GI higher than that of equivalent gluten-containing foods [50,51], although this is refuted by some authors [52]. Gluten-free foods have a higher GI, because gluten protein does not allow the easy access of amylase to hydrolyze starch granules in the lumen of the small intestine [50]. However, many foods with a high GI have been shown to only slightly increase blood glucose and vice versa [51,53]. Thus, the GI provides a measurement of the quality but not the quantity of the carbohydrates consumed. Meanwhile, blood glycemia is influenced by the synergistic interaction between the quantity and quality of carbohydrates. Therefore, epidemiological studies are utilizing a new concept to assess outcomes as a result of glucose metabolism: the glycemic load (GL). The GL may be calculated with the product of GI (as a percentage) of available carbohydrates, representing both the quality and quantity of carbohydrates consumed. The GL may be interpreted as a measure of insulin requested in free-living conditions, because the amount of carbohydrates consumed at each meal usually varies in such conditions [51,54,55]. Nevertheless, if the blood glucose response to food is a determinant of body weight remains controversial [56].

Several studies conducted in overweight or obese children show discordant results regarding the associations of GI and GL with obesity. One cross-sectional study reports no association of body fat with GI or GL [57]. Others studies show positive associations of GI and GL with waist circumference, BMI, and the sum of 4 skinfolds [58,59]. However, other cohort studies report inconclusive results [60,61,62]. A meta-analysis [63] that identified six eligible randomized clinic trials including a total of 202 participants concludes that low-GI or low-GL diets confer marked benefits on weight, BMI, total fat mass, and lipid profile. Regardless, further research on long-term improvements is required. A more recent systematic review provides evidence that long-term interventions with a low-GI/GL diet confer beneficial effects on fasting insulin and pro-inflammatory markers such as C-reactive protein; such interventions might prove to be helpful in the primary prevention of obesity-associated diseases [64]. These aspects could help explain the occurrence of overweight/obesity in celiac patients on a GFD.

On the other hand, several studies evaluating the effects of a GFD on metabolic control, growth, and nutritional status in celiac patients with type I diabetes provide a natural model of the interactions between diet, glycemic response, and nutritional status, demonstrating how this interrelationship can be much more complex. However, these studies have completely discordant results. Some studies [65] indicate improvements in BMI and glycosylated hemoglobin (HbA1c) levels in patients with CD and type I diabetes on a GFD. Meanwhile, Novòa Medina *et al*. [66] report no effect on the metabolic control, height, or weight of such patients. Other studies evaluated the influence of GFDs on metabolic parameters including insulin dose, HbA1c, glucose excretion, and hypoglycemic episodes. Saadah *et al*. [67] report that a GFD resulted in a significant improvement of growth and influenced diabetic control, particularly higher insulin levels in patients with CD than the levels at baseline. Other authors [68,69] found no significant difference in the insulin dose, HbA1c, 24-h urinary glucose excretion, or the number of hypoglycemic episodes. Abid *et al*. [70] found that a GFD reduced gastrointestinal symptoms in the short term and particularly episodes of severe hypoglycemia in children with type I diabetes with CD; however, there were no changes in the standard deviation scores for height, weight, BMI, or the average HbA1c before and after GFD consumption. Furthermore, epidemiological studies show energy intake is predictor of weight gain [71]. Thus, the GI and GL of the previous meal can theoretically influence energy intake in the next meal. A recent meta-analysis on this topic suggests that the GI, but not the GL might influence the energy intake of the next meal [72]. This may be because low-GI foods result in sustained blood glucose levels and hunger is delayed as compared with that after a high-GI meal [73]. Furthermore, recent evidence suggests energy intake is associated with changes in the resting metabolic rate [74]. The mechanism involved in this phenomenon may be the specific effect of blood glucose level on satiety (*i.e*., the glucostatic theory) or of other stimuli (e.g., peptides) involved in the control of appetite. Insulin and glucose stimulate the release of the leptin hormone that produces satiety and suppress the release of the ghrelin hormone that stimulates the appetite. Regardless, this does not precisely characterize relationships among GI, satiogenic leptin, and appetitic ghrelin. Furthermore, several gastrointestinal hormones called incretins are involved in the physiological control of hunger and satiety; they are involved in glucose metabolism and can act on pancreatic beta cells to stimulate insulin secretion. Among these hormones there is glucagon-like peptide-1 (GLP-1), which acts directly on the central nervous system and indirectly by slowing gastric emptying, inhibiting appetite and food intake, and inducing body weight reduction. The stimulation of insulin secretion by incretins is typically glucose dependent and manifests when glycemic levels are high but not when normal or low. Therefore, incretins have the potential to reduce hyperglycemia without causing hypoglycemia. A recent study revealed children with CD have a secretion pattern of gut-brain axis hormones that differs from that of controls. Alterations in this axis were more pronounced in children with both CD and type I diabetes mellitus; nevertheless, the roles of these gut-brain axis hormones in food intake and glycemic control in patients with CD and type I diabetes mellitus must be clarified [75].

Overall existing clinical evidence explains the variability of the anthropometric trends in CD patients after gluten withdrawal. However, it does not clarify why some CD patients develop overweight/obesity after beginning a GFD. Furthermore, as is the case in newly diagnosed CD patients, the global trend toward increased overweight/obesity could explain why CD patients on a GFD may become overweight. The changes in nutritional habits that induce the development of obesity are probably shared by CD patients and the general population. Table 2 summarizes the main pathogenetic links between CD and overweight/obesity.

**Table 2:** Suggested pathogenetic links between CD and overweight/obesity.

Time of Overweight/Obesity Diagnosis in Celiac Patients	Pathogenetic Link	Reference
**Overweight/Obesity at CD Presentation**	“Compensatory hypothesis”: high energetic yield due to the slow adaptation of the atrophic mucosa	[13]
	Global trend toward overweight/obesity in children	[39]
	Diagnosis not based on clinical symptoms but on screening test	[25,26]
**Overweight/Obesity on a GFD**	“Compensatory hypothesis”: normalization of caloric balance due to the restoration of mucosal functions	[13]
	Worldwide trend toward overweight/obesity in children	[39]
	Unpalatability of gluten-free foods, prompting the consumption of foods with high caloric content (*i.e*., fat and protein).	[20,29,40]
	High GI/GL of GFD?	[49,50,51,52,53,54,55,56,57,58,59,60,61,62,63,64,65,66,67,68,69,70,71,72,73,74]
	Altered secretion of gut–brain axis hormones?	[75]

CD: celiac disease; GFD: gluten-free diet; GI: glycemic index; GL: glycemic load.

## 4. Conclusions

Overweight/obesity is more common in children with CD than previously recognized. The prevalence of overweight in CD patients at diagnosis ranges from 8.8% to 20.8% [21,24,25,26,27], whereas that in CD patients on a GFD ranges from 9.4% to 21% [23,24,25,26]. Meanwhile, the prevalence of obesity in CD patients at diagnosis ranges from 0% to 6% [21,22,23,24,25,26,27], whereas that in CD patients on a GFD ranges from 0% to 8.8% [22,24,25,26,27]. Overweight/obesity is more frequent in newly diagnosed CD patients diagnosed on the basis of abdominal pain [22,26] and on the basis of screening procedures [25,26]. During follow-up, it is possible the unpalatability of gluten-free foods leads a preference for foods with high caloric fat and protein contents. However, the occurrence of overweight may be explained by the global trend toward overweight/obesity in children [39] including CD patients. An unconfirmed but nonetheless interesting hypothesis is that the development of overnutrition status is due to the compensatory high energetic yield secondary to the slow functional adaptation of the atrophic mucosa [13]. Therefore, mounting evidence suggests CD should be considered even in overweight/obese children in appropriate clinical settings.
